# Selection of treatment strategies for lumbar Brucella spondylitis: a retrospective clinical study

**DOI:** 10.3389/fsurg.2024.1365498

**Published:** 2024-03-22

**Authors:** Changhao Liu, Qiang Liu, Jianping Zheng, Ningkui Niu, Jiandang Shi, Zongqiang Yang

**Affiliations:** ^1^ Department of Orthopedics, Zhangye People’s Hospital Affiliated to Hexi University, Zhangye, Gansu, China; ^2^ The First Clinical Medical College of Ningxia Medical University, Yinchuan, Ningxia, China; ^3^ Department of Orthopedic, General Hospital of Ningxia Medical University, Yinchuan, Ningxia, China

**Keywords:** Brucella spondylitis, lumbar, treatment modalities, therapeutic efficacy, treatment strategy evaluation

## Abstract

**Objective:**

This study aims to investigate the treatment strategies for lumbar brucellar spondylitis by comparing the outcomes of pure pharmacological treatment with diseased intervertebral fixation fusion, with or without lesion clearance.

**Methods:**

A total of 157 patients with lumbar brucellar spondylitis were categorized into three groups: Group A (52 cases) received pure pharmacological treatment, Group B (53 cases) underwent posterior vertebral fixation fusion, and Group C (52 cases) received posterior (or anterior) lesion clearance followed by posterior vertebral fixation fusion. Clinical data were analyzed, and the efficacy of the three treatment methods was evaluated.

**Results:**

The surgical groups showed better outcomes at various time points compared to the pharmacological treatment group (*P* < 0.05). The pure fixation group outperformed the lesion clearance fusion group during the perioperative period (*P* < 0.05). The ESR, CRP, ODI scores, imaging evaluation and complications of the lesion clearance followed by fixation group were all better than those of the other two groups (*P* < 0.05). Surgical treatment groups showed no statistically significant difference in VAS scores (*P* > 0.05), and both were superior to the pharmacological treatment group. There were no statistically significant differences in clinical efficacy among the three groups at the last follow-up.

**Conclusion:**

Surgical treatment achieves early recovery goals compared to pharmacological treatment for brucellar spondylitis. However, individualized treatment principles should guide surgical decisions to select the most suitable approach for patients.

## 1 Introduction

Brucellosis, a bacterial disease caused by various Brucella species, is one of the most common zoonotic infections in the world. It is a multisystemic, systemic disease involving immune mechanisms and can lead to a variety of complications. It can invade the bones and joints, leading to infectious lesions. Brucella spondylitis (BS) is one of the serious complications of Brucella invasion of the spine, with a prevalence of 2%–65%, most often involving the lumbar spine (1, 2). Its clinical manifestation is a chronic infectious disease characterized by lower back pain, recurrent fever (usually intermittent), fatigue, excessive sweating, loss of appetite, and hepatosplenomegaly. The pathological basis is vertebral and intervertebral disc inflammation. But its clinical manifestation is atypical,and there is a lack of uniformity in the diagnosis and treatment, which can easily be misdiagnosed as spinal tuberculosis (3). Drug therapy is the basis of treatment for Brucella spondylitis, but the recurrence rate is 16%–29% (4) and there is still debate regarding the optimal treatment regimen and duration for brucella spondylitis. If there are symptoms such as spinal instability, kyphosis deformity, neurological compression, or uncontrollable pain caused by the lesions, drug treatment may not significantly alleviate the symptoms. In such cases, surgical treatment can significantly improve symptoms, effectively control the progression of the disease, increase the cure rate, and reduce relapse (5, 6). Therefore, this study analyzed clinical data of pure drug treatment, drug treatment-based posterior-only vertebral fusion, and posterior or anterior lesion clearance fusion in the treatment of lumbar brucella spondylitis. The aim was to evaluate the clinical efficacy of these three treatment methods for lumbar brucella spondylitis and explore surgical treatment strategies for lumbar brucella spondylitis, providing a theoretical basis for selecting different treatment methods.

## 2 Materials and methods

### 2.1 General clinical information

**Figure 1:**
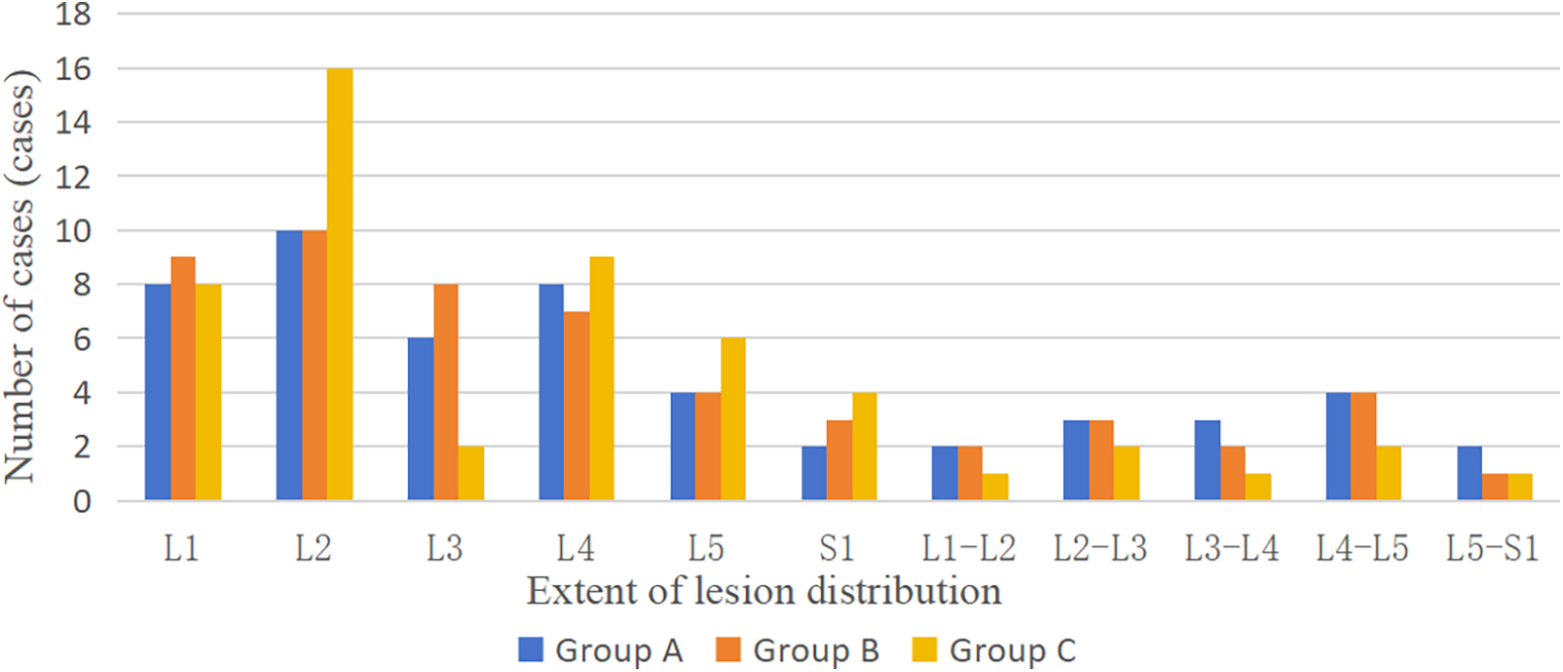
Distribution of the spinal brucobacterial spondylitis lesions in 157 patients.

In this study, we retrospectively analyzed the clinical data of 157 patients with lumbar brucella spondylitis treated in our department from January 2014 to December 2019. According to the treatment methods, they were divided into three groups: Group A (pure drug treatment group) with 52 cases, CT showed vertebral destruction in 7 cases (13.5%), accompanying paravertebral abscess in 10 cases (19.2%), endplate dissolution or sclerosis in 42 cases (80.8%), accompanying kyphosis deformity in 3 cases (5.7%), and lateral curvature deformity in 2 cases (3.8%); Frankel classification of neurological function: Grade D in 8 cases (13.8%), Grade E in 44 cases (84.6%); Group B (posterior vertebral fixation fusion + drug treatment group) with 53 cases, CT showed vertebral destruction in 10 cases (18.9%), accompanying paravertebral abscess in 14 cases (26.4%), endplate dissolution or sclerosis in 44 cases (83.0%), accompanying kyphosis deformity in 5 cases (9.4%), and lateral curvature deformity in 4 cases (7.5%); Frankel classification of neurological function: Grade D in 12 cases (22.6%), Grade E in 41 cases (77.4%); Group C [posterior vertebral fixation + posterior (or anterior) lesion clearance fixation fusion + drug treatment group] with 52 cases, CT showed vertebral destruction in 12 cases (23.1%), accompanying paravertebral abscess in 14 cases (26.4%), endplate dissolution or sclerosis in 45 cases (86.5%), accompanying kyphosis deformity in 6 cases (11.5%), and lateral curvature deformity in 4 cases (7.7%); Frankel classification of neurological function: Grade D in 12 cases (23.1%), Grade E in 40 cases (76.9%). The general clinical data of the patients in the three groups before treatment are shown in Table 1. The distribution of affected segments is shown in Figure 1. This research protocol was approved by the Ethics Committee of Ningxia Medical University General Hospital (approval number: KYLL-2021-676), and all patients signed informed consent forms.

**Table 1:** Comparison of the general clinical information of the three groups of patients.

Projects	Group A (52 Cases)	Group B (53 Cases)	Group C (52 Cases)	*F/X^2^*	*P*
Age (years)	52.38 ± 10.90	53.15 ± 11.22	51.50 ± 11.13	*F *= 0.29	*P *> 0.05
M/F (example)	32/20	33/20	31/21	*X^2 ^*= *0.08*	*P *> 0.05
Disease duration (months)	4.90 ± 3.50	5.11 ± 3.00	4.37 ± 2.64	*F *= 0.84	*P *> 0.05
Follow-up time (months)	22.81 ± 5.83	22.72 ± 5.84	24.83 ± 6.37	*F *= 2.05	*P* > 0.05
Symptoms and Clinical					
Back pain	48 (93.0)	49 (93.2)	48 (92.9)		
Intermittent fever	44 (83.7)	47 (88.6)	47 (90.5)		
Sensory impairment	41 (79.1)	43 (81.2)	41 (78.6)		
Fatigue	48 (93.0)	48 (90.9)	48 (92.9)		
Affected segments				–	–
Single segment	38 (72.9)	42 (79.5)	45 (86.5)		
Two segments	14 (27.1)	11 (20.5)	7 (13.5)		

### 2.2 Inclusion and exclusion criteria

#### 2.2.1 Inclusion criteria

(1) Standard tube agglutination test (SAT) titer ≥1:160, positive rose bengal plate test (RBT), (2) intractable low back pain with or without nerve or spinal cord compression, (3) abscess formation in the paravertebral area, lumbaris major muscle or spinal canal, (4) Bone destruction characterized by small cavities, limited amount of necrotic bone, or large necrotic bone and cavities, (5) Complete availability of all case data and follow-up information.

#### 2.2.2 Exclusion criteria

(1) Those with an unclear diagnosis or with other infectious or neoplastic diseases, (2) Follow-up period of less than 1 year or incomplete follow-up data, (3) Concomitant severe underlying diseases, (4) Inability to adhere to long-term follow-up.

### 2.3 Treatment methods

According to the treatment approach, patients were divided into three groups: Group A (52 cases) received drug therapy alone, Group B (53 cases) underwent posterior spinal fusion combined with drug therapy, and Group C (52 cases) underwent posterior spinal fusion with lesion clearance and fixation (via posterior or anterior approach) combined with drug therapy.

#### 2.3.1 Drug treatment group

Combined administration: oral doxycycline 100 mg/dose, 2 times/day + rifampicin 600 mg/dose, 1 time/day, 6 weeks as a course of treatment, The minimum duration of medication was 6 months, with adjustments made based on the patient's clinical manifestations, laboratory tests (normalization of ESR and CRP), and imaging evaluations. If necessary, the treatment duration could be extended up to 18 months (7).

#### 2.3.2 Surgical treatment group

The surgical treatment group consisted of two subgroups: Group B received posterior spinal fusion (posterior pedicle screw internal fixation and posterior lateral bone fusion), and Group C underwent posterior spinal fusion with lesion clearance and fixation (one-stage posterior pedicle screw internal fixation, lesion clearance, and posterior lateral bone fusion). All surgeries were performed under the guidance of the same responsible physician. All patients in the surgical treatment group received preoperative combined medication, including oral doxycycline 100 mg twice daily + rifampicin 600 mg once daily. Preoperative anti-Brucella treatment lasted for 3 weeks until the erythrocyte sedimentation rate (ESR) was ≤30 mm/1 h and C-reactive protein (CRP) was ≤45 mm/1 h with a decreasing trend. Then the surgery was performed.

(1) Posterior pedicle screw internal fixation and posterior lateral bone fusion

After general anesthesia, the patient was placed in a prone position. A long midline incision was made centered on the affected spinous process. The lumbar and back fascia were incised bilaterally, and the paraspinal muscles were dissected from the bone periosteum at the base of the spinous process. This exposed the spinous process, lamina, facet joints, and transverse process. Under C-arm fluoroscopy, pedicle screw internal fixation was performed. Before fixation, the intervertebral lamina of the affected segment was decorticated. Homologous allograft bone was implanted between the lamina, spinous process, and facet joints to promote fusion. Hemostasis was carefully ensured, and a drainage device was placed before closing the incision.

(2) Complete lesion clearance via posterior approach

After decompression of the lamina, the affected segment was exposed. After the placement of pedicle screws, the spinal cord, dura mater, and nerve roots were carefully examined. Pus within the spinal canal and inflammatory granulation tissue in the surrounding tissues were removed. A bone knife was used to clear the destroyed bone tissue, necrotic tissue of intervertebral discs, or damaged cartilage plates. After lesion clearance, the size of the bone graft bed was measured, and an appropriately sized autogenous iliac bone with three cortical surfaces was selected for interbody support. Finally, internal fixation was completed to correct deformities, and allograft bone was used for posterior lateral fusion. All specimens were sent for pathological examination and bacterial culture.

(3) Lesion clearance via anterior approach

The choice of anterior surgical approach depended on the location and extent of the Brucella infection. For the thoracolumbar segment, a combined thoracoabdominal or retroperitoneal approach was used. For the upper lumbar vertebrae, a lateral decubitus position with a flank incision was used. For the lower lumbar and sacral vertebrae, a supine position with an inverted “8” incision and retroperitoneal approach were used. The approach was selected based on the severity of vertebral destruction and the size of the abscess. Layer by layer, the abscess was exposed. A large needle was initially used to accurately locate the abscess, followed by an enlargement of the puncture site. The pus was aspirated using a suction device, and the abscess cavity was opened. The pus and caseous material were scraped off, and the fistula tract was identified. Along the fistula tract, the affected vertebra was located. Vascular segments supplying the affected vertebra were ligated, and the affected intervertebral disc and damaged vertebral body were thoroughly exposed and excised. A bone knife or curette was used to remove the lesion from the edge toward the periphery until a “sub-normal bone” state was achieved, characterized by a granular appearance without sclerosis, sequestra, caseous material, or granulation tissue, revealing fresh bone surfaces. After complete lesion clearance, the wound was repeatedly irrigated with saline. The size of the bone graft bed was measured, and an appropriately sized autogenous iliac bone with three cortical surfaces was selected for interbody support.

### 2.4 Post-operative management

Standard orthopedic care for the spine, enhanced nutrition. When the postoperative drainage volume is less than 50 ml/24 h, the drainage tube is removed. After 2–4 weeks of bed rest, the patient is allowed to engage in limited activity while wearing a lumbar brace. All patients continue to take doxycycline 100 mg per dose, twice daily, plus rifampicin 600 mg per dose, once daily. Liver and kidney function, complete blood count, C-reactive protein, erythrocyte sedimentation rate, and standard tube agglutination test for Brucella are regularly monitored monthly, with the addition of hepatoprotective drugs if necessary. Medication is discontinued when the erythrocyte sedimentation rate, C-reactive protein, and two consecutive months of negative standard tube agglutination test results are within the normal range.

### 2.5 Observation and evaluation indicators

(1) Perioperative evaluation indicators: The surgical group observes the operation time, intraoperative blood loss, postoperative drainage volume, and whether blood transfusion is required for surgical patients.
(2) Evaluation of patients: Pain visual analog scale (VAS), Oswestry Disability Index (ODI), erythrocyte sedimentation rate (ESR), C-reactive protein (CRP), and standard tube agglutination test results are assessed before treatment, at 1, 3, and 6 months after treatment, and at the last follow-up.
(3) Radiographic evaluation: Blind assessment of radiographic scores before treatment, at 1, 3, and 6 months after treatment, and at the last follow-up. The radiographic assessment includes x-ray evaluation of spinal stability, CT evaluation of abscess disappearance or calcification, assessment of lesion margin clarity and bone repair, and MRI evaluation of spinal recovery at 6 and 12 months postoperatively. The blind assessment of MRI results is quantitatively scored, with 2 points each for lesion area repair, stability, perispinal tissue changes, nerve compression, and degree of bone destruction, totaling 10 points, with 0 points indicating no improvement.
(4) Clinical Efficacy Evaluation and Complications: Clinical efficacy is evaluated on the following criteria (8): ①Cure: Normal body temperature, complete relief of lumbago or VAS score of 0, complete restoration of daily activity ability, Frankel E-grade for neurological function, imaging showing absorption of vertebral inflammation and infection and spinal stability, ESR and CRP within the normal range, negative standard tube agglutination test (SAT). ②Improvement: Normal body temperature, partial relief of lumbago: significant improvement ≥50% compared to before treatment or VAS score of 1–4, partial restoration of daily activity ability (≥50%), imaging showing partial absorption of vertebral inflammation and repair of bone destruction, decreased ESR and CRP, negative SAT. ③Ineffective: Some relief of lumbago (<50%) or VAS score above 5, poor or no recovery of daily activity ability (<50%), no improvement in vertebral inflammation, no significant change in ESR and CRP compared to before treatment, positive SAT, recurrence after discontinuation of treatment for 2 weeks.

### 2.6 Statistical methods

The SPSS25.0 software system was used for analysis, and the measurement data conforming to a normal distribution were expressed as mean ± standard deviation (*`x ± s*). One-way ANOVA was used for measurement data conforming to normal distribution and Kruskal-Wallis H non-parametric test was used for non-conformity; chi-square test was used for counting data (X^2^). *α*=0.05, *P *< 0.05 indicated differences are statistically significant.

## 3 Results

### 3.1 Perioperative evaluation indicators

All patients had perfect follow-up data, with all three groups being followed up for >12 months. In the surgical treatment group, the posterior approach disease vertebral interbody fusion group had better results than the posterior approach disease vertebral interbody fusion combined with lesion clearance and fixation group in terms of surgical time, intraoperative blood loss, postoperative drainage volume, and blood transfusion. The differences were statistically significant (*P *< 0.05, Table 2).

**Table 2:** Comparison of perioperative evaluation indicators between the two groups $\left(\bar{x}\pm s\right)$.

Observation indicators	Group B (*n* = 53)	Group C (*n* = 52)	t/x^2^	*P*
Operating time(h)	2.04 ± 0.86	2.9 ± 1.06	*t* = 20.92	*P* < 0.01
Blood Loss (ml)	440.57 ± 136.71	524.23 ± 114.26	*t* = 11.56	*P* < 0.01
Postoperative drainage (ml)	67.36 ± 16.37	96.06 ± 23.89	*t* = 51.73	*P* < 0.05
Transfusion (yes/no)	13/40	30/22	*x*^2 ^= 11.94	*P* < 0.01

### 3.2 Evaluation of laboratory test results

Prior to treatment, all patients had positive Brucella agglutination tests (SAT), which turned negative at the last follow-up. The ESR and CRP results before treatment, at 1, 3, and 6 months, and at the final follow-up are presented in Tables 3, 4. The results indicated that there were no significant differences in infection markers among the groups before treatment (*P *> 0.05). At 1, 3, and 6 months after treatment, the surgery group (Group A and Group B) showed significantly lower ESR and CRP levels compared to the medication group (*P *< 0.05). However, at the last follow-up, all groups had infection markers within the normal range, and the differences were not statistically significant (*P *> 0.05). The lesion clearance and fixation group showed faster recovery of ESR and CRP levels at 1 and 3 months after treatment compared to the fixation-only group (*P *< 0.05).

**Table 3:** Comparison of ESR (mm/h) scores before and after treatment $\left(\bar{x}\pm s\right)$.

Group	Cases	Before treatment	1 Month	3-Month	6-Month	Last follow- up
Group A	52	45.31 ± 11.67	38.00 ± 3.83	24.94 ± 2.49	13.12 ± 8.64	4.63 ± 2.37
Group B	53	45.23 ± 23.89	31.83 ± 5.17*^,^^#^	12.77 ± 3.32*^,^^#^	9.17 ± 7.62*	4.42 ± 3.28
Group C	52	45.31 ± 23.98	28.57 ± 4.75*	11.29 ± 4.25*	9.19 ± 8.40*	4.12 ± 2.61
*F*		0.02	56.42	248.98	3.78	0.46
*P*		>0.05	<0.01	<0.01	<0.05	>0.05

Normal ESR range: male: 0–15 mm/h, female: 0–20 mm/h.

* *P *< 0.05 compared to drug treatment group.

^#^ *P *< 0.05 compared to lesion clearance group.

**Table 4:** Comparison of CRP(mg/L) scores before and after treatment $\left(\bar{x}\pm s\right)$.

Group	Cases	Before treatment	1 Month	3-Month	6-Month	Last follow- up
Group A	52	38.94 ± 17.37	28.78 ± 4.65	19.51 ± 2.49	4.76 ± 2.20	2.97 ± 2.54
Group B	53	38.72 ± 12.46	22.47 ± 6.00*^,^^#^	14.26 ± 6.44*^,^^#^	3.69 ± 2.19*	2.16 ± 1.92
Group C	52	38.65 ± 23.74	20.72 ± 4.33*	10.55 ± 3.07*	3.40 ± 2.09*	2.44 ± 2.13
*F*		0.017	16.49	43.61	5.78	2.49
*P*		>0.05	<0.01	<0.01	<0.01	>0.05

CRP Normal value range: 0–10 mg/L.

* *P*<0.05 compared to drug treatment group.

^#^ *P *< 0.05 compared to lesion clearance group.

### 3.3 Evaluation of VAS and ODI improvements

The results showed that there was no statistically significant difference in VAS and ODI scores among the three groups before treatment (all *P *> 0.05). After treatment, there was a significant reduction in pain and improvement in self-care ability in all groups within the first week (*P *< 0.05) (Tables 5, 6). At the last follow-up, it was found that the VAS scores in the surgical treatment group were superior to those in the medication group at all time points, with statistical significance (*P *< 0.05). There was no statistically significant difference in VAS scores between the pure fixation group and the lesion clearance with fixation group at all time points (all *P *> 0.05). At one week after treatment, the ODI score improvement in the lesion clearance with fixation group was superior to that in the pure fixation group (*P *< 0.05), but at the last follow-up, there was no statistically significant difference in ODI scores among the three groups (all *P *> 0.05, Tables 5, 6).

**Table 5:** Comparison of VAS scores before and after treatment $\left(\bar{x}\pm s\right)$.

Group	Cases	Before treatment	1 Week	3-Month	6-Month	Last follow- up
Group A	52	8.29 ± 0.69	4.92 ± 0.99	3.42 ± 0.98	2.92 ± 1.53	1.10 ± 0.80
Group B	53	8.34 ± 0.65	3.23 ± 0.64*	1.58 ± 0.50*	1.36 ± 0.83*	0.64 ± 0.59
Group C	52	8.15 ± 0.67	3.37 ± 0.77*	1.54 ± 0.50*	1.62 ± 0.89*	0.83 ± 0.51
*F*		1.07	70.62	124.59	28.88	6.58
*P*		>0.05	<0.01	<0.01	<0.05	<0.05

* *P *< 0.05 compared to conservative treatment group.

**Table 6:** Comparison of ODI scores before and after treatment $\left(\bar{x}\pm s\right)$.

Group	Cases	Before treatment	1 Week	3-Month	6-Month	Last follow- up
Group A	52	74.62 ± 2.87	66.69 ± 4.31	52.58 ± 3.50	32.94 ± 7.72	4.58 ± 1.78
Group B	53	74.11 ± 3.11	47.70 ± 3.78*^,^^#^	30.13 ± 4.12*	16.04 ± 5.17*	4.75 ± 2.12
Group C	52	74.11 ± 3.11	47.70 ± 3.78*^,^^#^	30.13 ± 4.12*	16.04 ± 5.17*	4.75 ± 2.12
*F*		0.39	289.80	487.33	150.79	0.10
*P*		>0.05	<0.01	<0.01	<0.01	>0.05

* *P *< 0.05 compared to conservative treatment group.

^#^ *P *< 0.05 compared to lesion clearance group.

### 3.4 Evaluation of imaging

**Figure 2:**
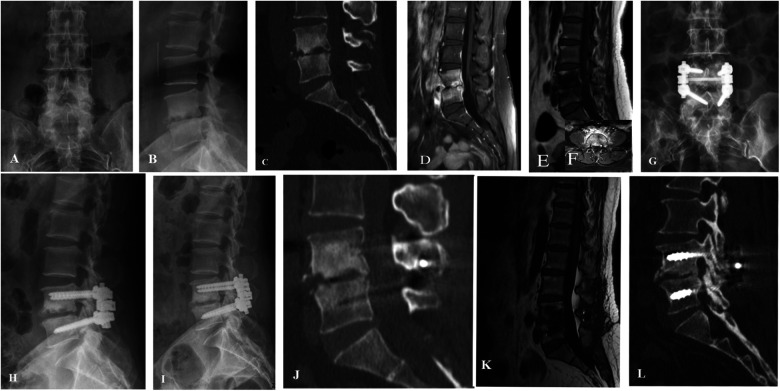
Patient, female, 52 years old, diagnosed with brucellar spondylitis at L4∼5 level, underwent a procedure of posterior instrumentation with posterior lateral interbody fusion. (**A, B**) Preoperative lumbar spine anteroposterior and lateral x-rays show narrowed L4∼5 disc space with adjacent endplate sclerosis, blurring, and irregular margins, accompanied by osteophyte formation, and a loss of normal lumbar lordosis. (**C**) Preoperative lumbar spine CT reveals L4 vertebral body inferior margin hypertrophy and sclerosis coexisting with osteolytic lesions. Localized sclerosis at the vertebral body edges and multiple areas of bone destruction are evident, with more pronounced peripheral involvement. (**D, E**) Preoperative lumbar spine MRI in sagittal T1-weighted and T2-weighted images show narrowed L45 disc space, decreased signal intensity in L45 vertebral bodies, endplate erosion, and disc space narrowing. (**F**) Preoperative lumbar spine MRI in transverse T2-weighted image indicates an epidural abscess in front of the dural sac and a paraspinal abscess, with multiple areas of high signal intensity representing inflammatory tissue within the vertebral bodies. (**G, H**) Postoperative x-rays at 1 week demonstrate satisfactory positioning of the internal fixation and restoration of intervertebral height. (**I, J** and **K**) Postoperative x-rays at 3 months reveal well-positioned internal fixation and satisfactory intervertebral fusion, with significant absorption of the epidural and paraspinal abscesses compared to before. (**L**) Postoperative CT at 6 months shows satisfactory interbody fusion with formation of bone bridges at the anterior and posterior edges of the vertebral bodies, indicating successful healing.

**Figure 3:**
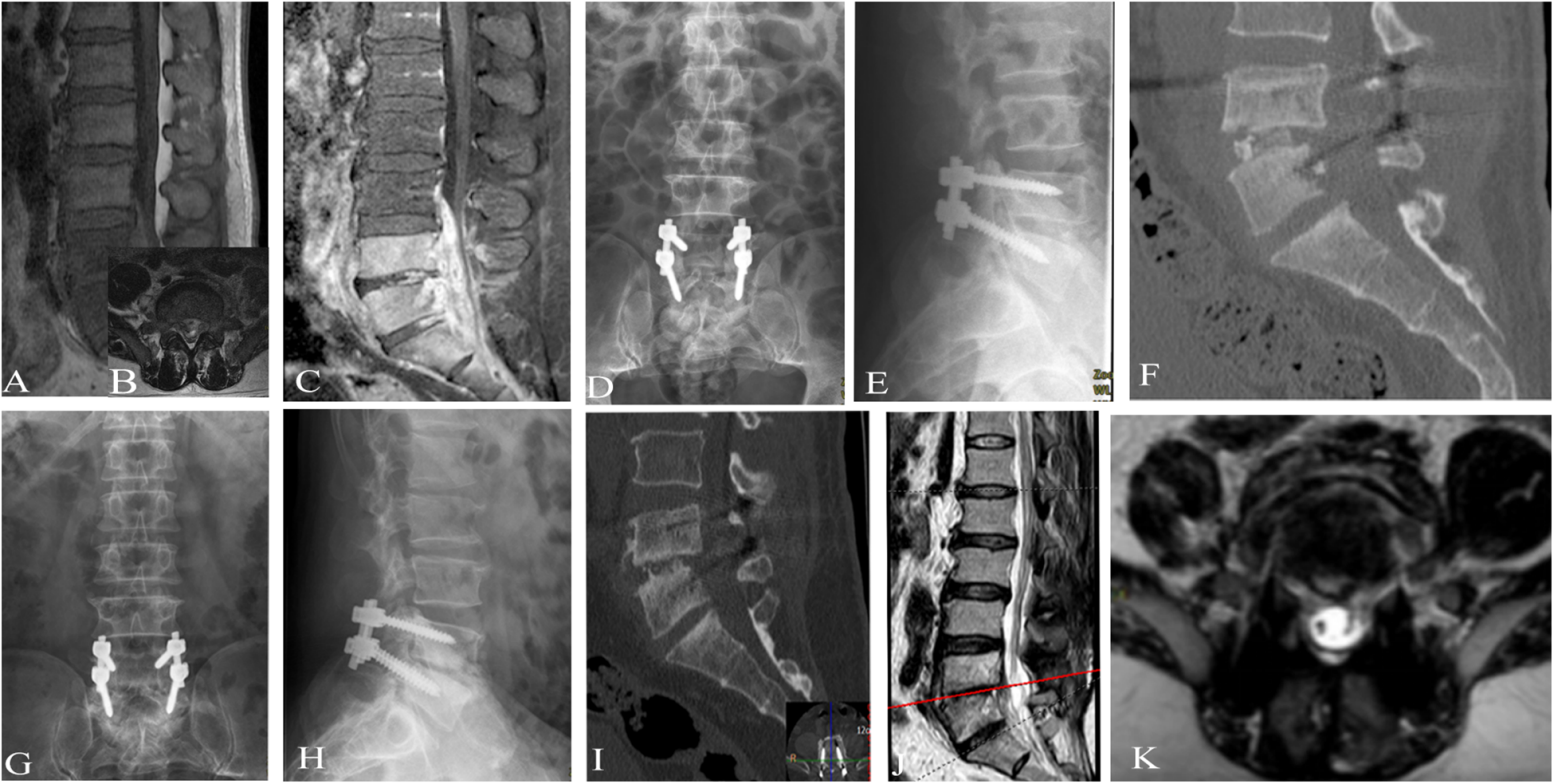
Patient, male, 42 years old, diagnosed with brucellar spondylitis at L4∼5 level, underwent a procedure of posterior instrumentation with clearing of the lesion and interbody fusion. (**A**) Preoperative MRI in sagittal view reveals inflammatory infiltration and localized destruction of the vertebral body with mixed long T2 signal intensity. (**B**) Preoperative MRI in sagittal view shows vertebral body and intervertebral disc destruction, along with an intervertebral space abscess and an extradural abscess compressing the corresponding dural sac, appearing as non-uniform high signal intensity on T2-weighted imaging. (**C**) Preoperative MRI in transverse view shows formation of paraspinal and extradural abscesses, with compression of the dural sac. (**D, E**) Postoperative x-rays on the first day demonstrate complete decompression of the L4∼5 vertebral space with satisfactory positioning of the internal fixation. (**F**) Postoperative CT on the first day shows clearance of the lesion and interbody fusion at the 4∼5 space. (**G, H**) Postoperative x-rays at 6 months show well-positioned internal fixation and successful interbody fusion. (**I**) Postoperative CT at 6 months indicates clear visibility of the 4 vertebral lesion's edge with repaired bone quality and reduced necrotic bone fragments, and formation of a bone bridge at the anterior edge of the 4 vertebral body. (**J, K**) Postoperative MRI at 6 months reveals absorption of the intervertebral space infection and extradural abscess, along with repaired bone destruction, well-aligned spinal nerves in the dural sac, and stable spine.

**Figure 4:**
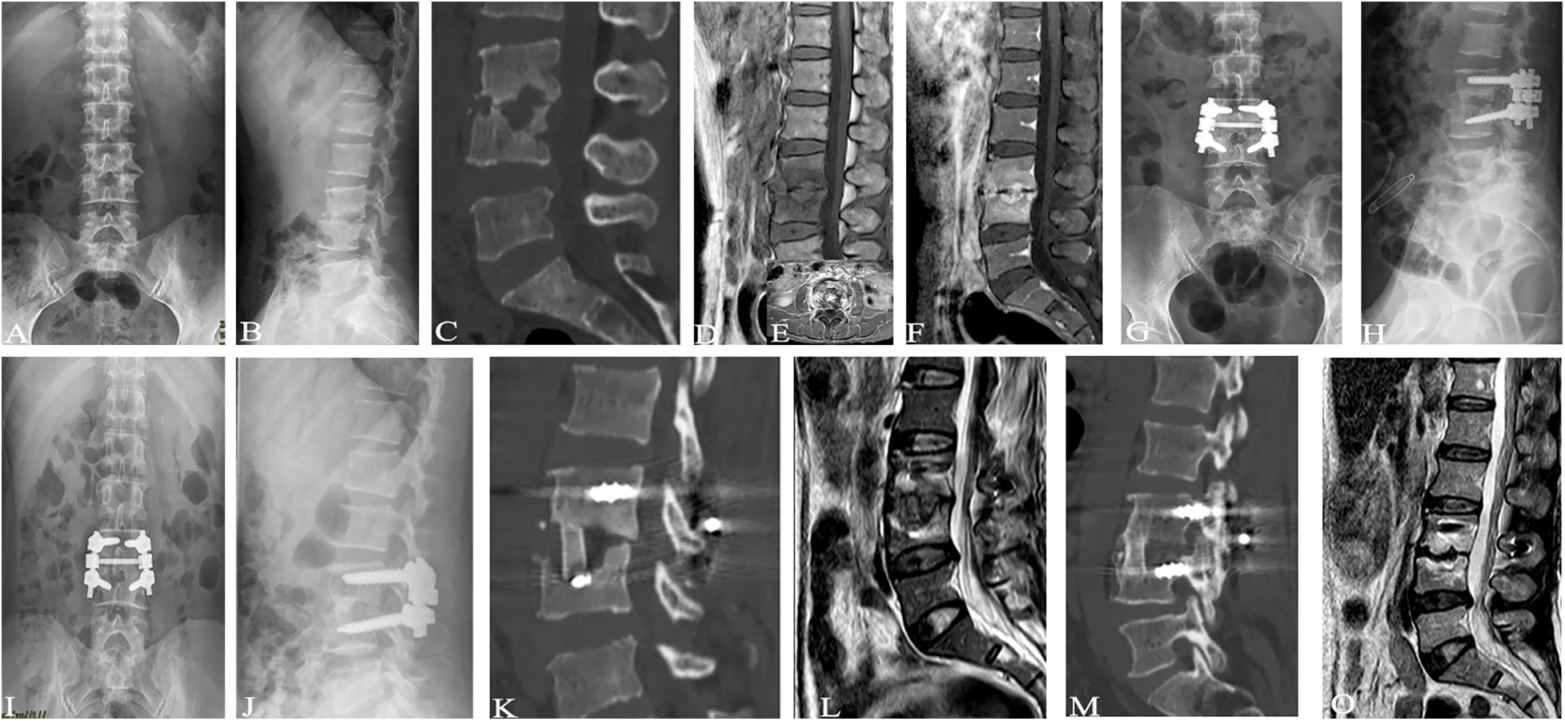
Patient, female, 54 years old, diagnosed with brucellar spondylitis at L3∼4 level, underwent a procedure of posterior instrumentation with anterior lesion clearance using iliac bone fusion. (**A, B**) Preoperative lumbar spine anteroposterior and lateral x-rays show narrowing of the L3∼4 disc space, formation of a bony bridge between the vertebral bodies, and partial bone destruction at the lower border of L3 and upper part of L4, with the presence of cavities and necrotic bone formation. (**C**) Preoperative lumbar spine CT reveals significant narrowing of the L3∼4 disc space, with evident cavity-like destruction in the vertebral bodies. (**D**–**F**) Preoperative lumbar spine MRI indicates the formation of abscesses in the psoas muscle and intraspinal area, with compression of the dural sac. (**H**, **I**) Postoperative x-rays on the first day show complete decompression of the vertebral plates, restoration of vertebral height, and proper positioning of the L3∼4 vertebral body graft. (**J**) Postoperative CT on the first day demonstrates successful fixation and interbody fusion at L3∼4. (**K**) Postoperative MRI on the first day reveals clearance of the lesions in the vertebral bodies and surrounding areas, with bone grafting in the L3∼4 space. (**L, M**) Postoperative x-rays at 4 months show well-positioned internal fixation and successful interbody fusion, with formation of a bone bridge at the anterior edge of the vertebral bodies. (**N**) Postoperative CT at 4 months indicates clear visibility of the L4 vertebral lesion's edge, reduced cavity size, sclerosis, disappearance of necrotic bone fragments, and near-complete healing. (**O**) Postoperative MRI at 4 months shows no signs of inflammatory infiltration or bone destruction in the original lesion, no compression of the dural sac, and absorption of the paraspinal abscess, indicating a stable spine.

**Figure 5:**
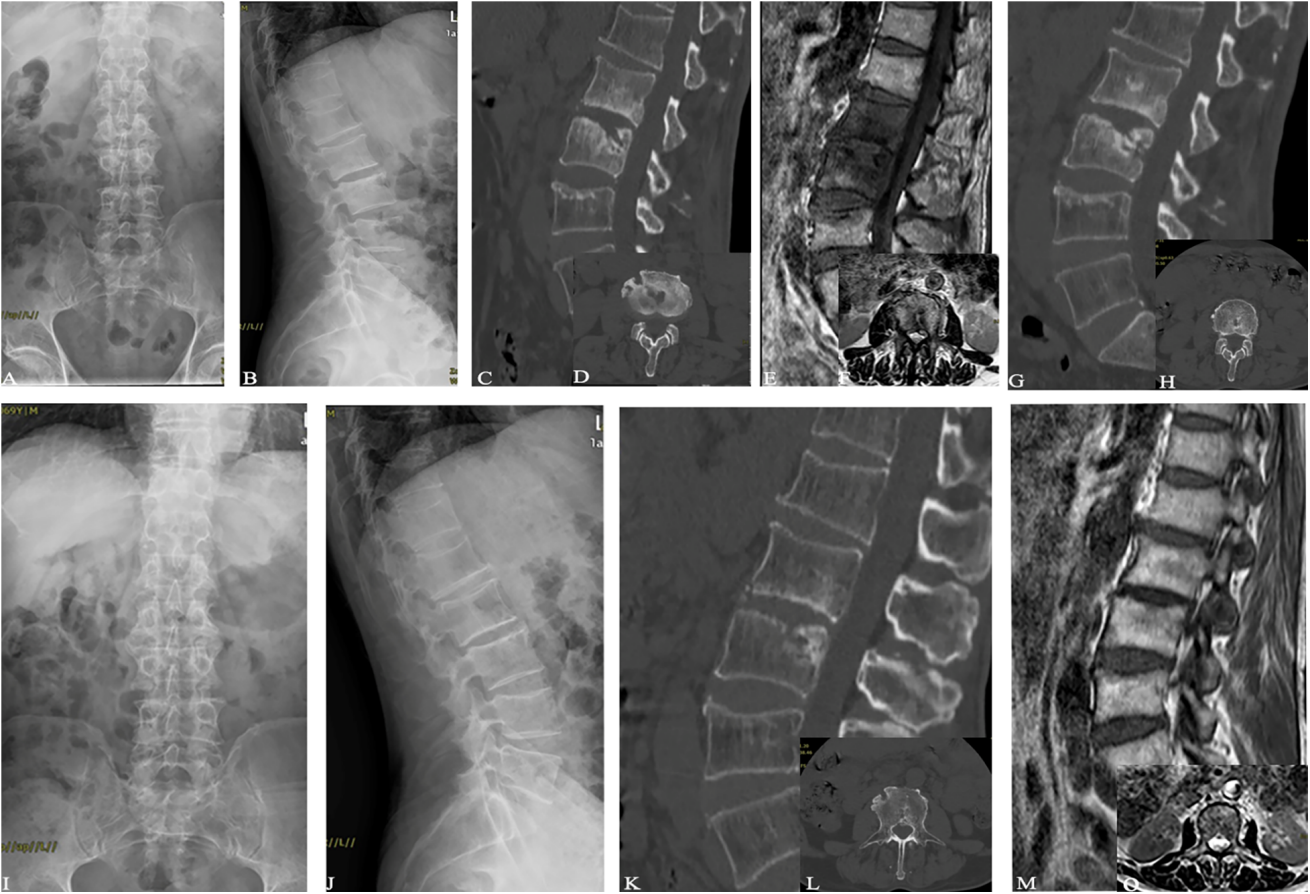
Patient,male, 67 years old, L2∼3 brucellosis spondylitis, conservative treatment with oral medication (**A, B**) before treatment, lumbar spine frontal-lateral radiographs showed narrowing of L2∼3 intervertebral space, with no obvious cavity and dead bone formation; (**C, D**) before treatment, lumbar vertebral CT showed narrowing of L2∼3 intervertebral space, and obvious cavity-like destruction of L3 vertebral body; (**E, F**) before treatment, lumbar vertebral MRI showed obvious destruction of vertebral body, with a small amount of abscess formation in the vertebral canal 4, 4, 3 months after treatment, lumbar CT showed that the dead bone was absorbed and the cavity-like destruction was reduced; (**I, J**) 6 months after treatment, lumbar x-ray showed that a bone bridge was formed at the posterior margin of the vertebral body; (**K, L**) 6 months after treatment, lumbar CT showed that the edges of the vertebral body lesions were clear, and the cavities were narrowed and sclerotic, with the absorption of the dead bone disappeared; (**M, N**) 6 months after treatment, lumbar MRI showed that there was no inflammatory infiltration and bone destruction in the vertebral body of the original lesion and no compression of the intravertebral dural sac, and the paraspinal dura was not compressed, and the paraspinal dural sac was not compressed. MRI of lumbar spine showed no inflammatory infiltration and bone destruction in the original lesion vertebral body, no compression of the dural sac in the spinal canal, absorption of paravertebral abscess, and stabilization of spine.

The blind evaluation scores of radiological observations in the three treatment groups showed improvement at each time point after treatment compared to before treatment (Figures 2–5). The lesion clearance and fixation group demonstrated significantly higher radiological scores than the pure fixation group at 1 and 3 months of follow-up after surgery (*P *< 0.05). However, there was no statistically significant difference in the scores among the three groups at the final follow-up (*P *> 0.05). No occurrences of loosening, fracture, or segmental collapse of internal fixation were observed during the follow-up period (Table 7).

**Table 7:** Comparison of blinded assessment scores for imaging observations before and after treatment $\left(\bar{x}\pm s\right)$.

Group	Cases	Before treatment	1 Month	3-Month	6-Month	Last follow- up
Group A	52	1.83 ± 1.08	2.35 ± 0.86	3.38 ± 1.16	4.92 ± 1.70	7.04 ± 1.46
Group B	53	1.92 ± 1.36	3.51 ± 1.03*^,^^#^	5.62 ± 1.04*^,^^#^	6.53 ± 1.12*	8.91 ± 1.01
Group C	52	2.12 ± 1.15	3.19 ± 0.99*	4.35 ± 0.76*	6.73 ± 1.19*	8.44 ± 2.10
*F*		0.78	20.37	66.00	27.61	19.77
*P*		>0.05	<0.01	<0.01	<0.01	<0.01

* *P* < 0.05 compared to the conservative treatment group.

^#^ *P* < 0.05 compared to the lesion clearance group.

### 3.5 Evaluation of clinical efficacy

According to the criteria for clinical efficacy evaluation, the outcomes gradually improved during the postoperative follow-up period (Table 8). At the 3-month follow-up after treatment, in Group A, 6 cases (11.54%) were cured, 30 cases (57.69%) showed improvement, and 16 cases (30.67%) were ineffective. In Group B, 22 cases (41.5%) were cured, 28 cases (52.83%) showed improvement, and 3 cases (5.57%) were ineffective. In Group C, 24 cases (46.15%) were cured, 26 cases (50.00%) showed improvement, and 2 cases (3.85%) were ineffective. At the 6-month follow-up after treatment, in Group A, 19 cases (36.54%) were cured, 28 cases (53.85%) showed improvement, and 5 cases (9.62%) were ineffective. In Group B, 45 cases (84.91%) were cured, 7 cases (13.21%) showed improvement, and 1 case (1.89%) was ineffective. In Group C, 40 cases (76.92%) were cured, 11 cases (21.15%) showed improvement, and 1 case (1.92%) was ineffective. At months 3 and 6, we compared the cure rates of the three groups and found that the cure rate was significantly higher in all surgical groups than in the drug-only group (*P *< 0.05). There was no statistically significant difference in the comparison between the fusion alone group and the lesion removal + internal fixation group (*P *> 0.05). At the last follow-up (time greater than 12 months), all patients were cured.

**Table 8:** Comparison of the outcome of the three groups of patients with Brucella spondylitis n(%).

Group	Csaes	3-Month Treatment				6-Month Treatment			
		Cure	Improvement	Ineffective	Cure rate	Cure	Improvement	Ineffective	Cure rate
Group A	52	6	30	16	11.54	19	28	5	36.54
Group B	53	22	28	3	41.51*^,^^#^	45	7	1	84.91*^,^^#^
Group C	52	24	26	2	46.15*	40	11	1	76.92*

* *P* < 0.05 compared to the conservative treatment group.

^#^ *P* > 0.05 Compared to the lesion clearance group at 3 months postoperative.

Compared to before treatment, at the last follow-up, the neurological function based on the Frankel grading scale showed the following distribution: In Group A, 6 cases (11.54%) were classified as Grade D, and 46 cases (88.46%) were classified as Grade E. In Group B, 2 cases (3.77%) were classified as Grade D, and 51 cases (96.23%) were classified as Grade E. In Group C, 1 case (1.92%) was classified as Grade D, and 51 cases (98.08%) were classified as Grade E. The difference compared to preoperative status was statistically significant (X^2 ^= 4.086, *P *< 0.05).

### 3.6 Complications

In the medication treatment group, 10 cases experienced severe lumbago and limited mobility, along with neurological impairment in both lower limbs. Symptomatic treatments such as anti-inflammatory drugs, pain relief, and lumbar immobilization were administered but provided limited relief. Surgical treatment was chosen for these 10 patients. They were excluded from the original study, and an additional 10 research cases were supplemented in the medication treatment group. Among these, 14 cases developed varying degrees of liver damage after 6 weeks of medication use. In the posterior approach spinal fusion group, two patients experienced incisional infection after surgery, which was treated with sensitive antibiotics based on bacterial culture and drug sensitivity tests. After 2 weeks of treatment and wound care, the infections healed. Three cases experienced gastrointestinal reactions. In the posterior approach spinal fusion and lesion clearance and fixation group (combined posterior and anterior approaches), two cases developed incisional infections, which healed after wound debridement, drainage, and 2 weeks of treatment. One case had a cerebrospinal fluid leak due to dural rupture during surgery. The dural tear was repaired during the operation, and good healing was achieved after postoperative bed rest without a pillow and thorough drainage. Five cases in this group experienced liver damage.

## 4 Typical case

## 5 Discussion

Currently, there is no standardized treatment approach for brucellar spondylitis. As it is an infectious condition caused by bacteria in the spine, pharmacological treatment is the fundamental and primary aspect, following the principles of “long-term, adequate dosage, combination therapy, and multiple administration routes.” The treatment regimen recommended by the World Health Organization (WHO) for brucellosis includes doxycycline 200 mg/day in combination with rifampicin 600–900 mg/day for 6 weeks, or doxycycline 200 mg/day (tetracycline 2 g/day) for 6 weeks plus streptomycin 1 g/day for 2–3 weeks (9). However, studies have reported an effectiveness rate of only 60% for this treatment regimen, with recurrence rates as high as 60% (10–12). Some studies have indicated that prolonging the duration of drug therapy can reduce the recurrence rate. However, currently, there is a lack of standardized and short-course treatment regimens similar to those used for tuberculosis. Pappas et al. (13) suggested that the treatment duration is the main factor influencing the effectiveness of brucellar spondylitis treatment, rather than specific recommended regimens. Moreover, the failure rate is much higher for treatment courses lasting 6 weeks or less compared to continuous treatment for over 12 weeks (43.66% vs. 17.43%). Therefore, the recommended treatment duration by the World Health Organization (WHO) for acute phase uncomplicated brucellar spondylitis is clearly insufficient. Based on our team's treatment experience, a combination of doxycycline and rifampicin for at least 6 months can significantly reduce the recurrence rate (14, 15). Ioannou S. suggested that a triple therapy (doxycycline + rifampicin, combined with streptomycin or compound sulfamethoxazole or ciprofloxacin) should be used for at least 6 months to achieve effective cure (7). Raptopoulou et al. (16) also found that even in cases of severe brucellar spondylitis, effective clinical control can be achieved through rational and long-term antibiotic treatment. In this study, it was observed that with the passage of time, the drug therapy group achieved normal values for various clinical indicators within 24–36 months, compared to the surgical treatment group, further confirming the aforementioned views. However, a problem exists in that drug therapy cannot provide rapid relief of lumbar pain symptoms or offer effective activity support to patients, leading to some patients eventually choosing surgical treatment.

Therefore, we believe that based on the imaging classification, long-term and rational drug therapy can achieve satisfactory therapeutic effects only in cases where the spine is stable, there is no inflammatory destruction of the intervertebral disc, vertebral body or small abscess, and there are no neurological impairments. However, the recurrence rate of pure drug therapy still accounts for 16%–29% (3), and long-term use of medications can cause liver and kidney damage and drug resistance, which imposes a considerable economic and physical burden on patients. For patients who cannot tolerate or use doxycycline treatment (e.g., during pregnancy), alternative options such as methoxybenzylpyridine or sulfamethoxazole can be used (17). In the end, among the 53 patients treated with pure drug therapy in this study, 14 patients experienced liver function damage, which was relieved after timely use of hepatoprotective drugs. Additionally, 10 patients experienced drug intolerance and intolerable pain, leading to the choice of surgical treatment. At the end of treatment, all patients showed relief of symptoms.

For patients who have not experienced pain relief after rigorous drug therapy, have severe vertebral destruction, and significant compression of the spinal cord and nerves, surgical treatment is necessary (18). The goal of surgery is to remove the lesions, relieve pain, restore spinal stability, and regain spinal and spinal cord function, ultimately achieving rapid recovery. Currently, the surgical approaches for treating lumbar brucellar spondylitis mainly include anterior approach, posterior approach, and combined anterior-posterior approach surgery. Among them, the posterior approach surgery allows direct exposure of the affected vertebra within a small incision, resulting in shorter operation time, less blood loss, and minimal impact on surrounding normal tissues. Therefore, it is relatively safe and can maintain posterior column stability with the dual assurance of the pedicle screw system in the short term and bone fusion in the long term. Hence, it has become the most widely used surgical approach nowadays (10, 19). In this study, 105 patients who met the indications for surgical treatment and underwent surgery were selected. After standardized drug therapy and relief of systemic toxic symptoms, patients were assessed preoperatively based on their imaging classification. The extent of vertebral destruction, size of paravertebral abscess, and degree of neurological impairment were observed. Surgical procedures included pure posterior spinal fixation with bone fusion and posterior spinal fixation combined with lesion clearance through a posterior-anterior approach. The postoperative follow-up results showed significant improvement in various clinical indicators in both groups during the follow-up period (*P *< 0.05), especially in terms of VAS score. Compared to the pure drug therapy group, the surgical group showed significant improvement immediately after surgery, with a cure rate of 100% and an improvement rate of 100%. Inflammatory markers such as ESR and CRP returned to normal range in both groups, and both groups achieved bone fusion and primary healing within 6–12 months after surgery, without postoperative complications such as reinfection or recurrence. When comparing the surgical time, intraoperative blood loss, and incidence of complications between the two groups, pure posterior spinal fixation with bone fusion was superior to lesion clearance and fixation with bone fusion (*P *< 0.05). However, there was no statistically significant difference in the cure rate (*P *> 0.05). This indicates that both surgical approaches can effectively treat lumbar brucellar spondylitis, but pure posterior spinal fixation with bone fusion has the advantages of less trauma and fewer postoperative complications.

In cases of brucellar spondylitis, there is localized destruction of the vertebral body with significant residual bone sclerosis. The presence of local lesions and surrounding sclerotic bone provides sufficient grip for internal fixation devices after complete lesion clearance. Additionally, the bone destruction in brucellar spondylitis primarily occurs at the anterior edge of the vertebral body, preserving the integrity of the vertebral body morphology to a large extent. This provides an anatomical basis for lesion clearance and internal fixation through a posterior approach. Moreover, in brucellar spondylitis, there is simultaneous new bone formation and local inflammatory reaction within the vertebral lesions. The vascularity is abundant, making it difficult for sclerosis to form, and antimicrobial drugs can easily penetrate the lesion area. Furthermore, Brucella has low invasiveness and is sensitive to drug treatment. These factors serve as therapeutic guarantees for pure posterior spinal fixation and fusion, and the restoration of spinal stability. On the basis of rigorous drug therapy, continuing the use of anti-brucellosis drugs for 7–8 months after surgery can achieve the desired treatment outcomes. Summarizing the two surgical approaches, we found that regardless of lesion clearance, patients experienced significant relief of lower back pain after pedicle screw fixation and bone grafting. The main reasons for this are as follows: on one hand, the local instability of the affected lumbar segment and increased pressure within the intervertebral disc are the main causes of severe lower back pain (20). After internal fixation, the pressure on the vertebral bodies and intervertebral discs is released, and the local spinal stability is restored. On the other hand, the abscess in lumbar brucellar spondylitis is small and localized, resulting in the release of a large amount of inflammatory factors confined to the lesion area, causing continuous stimulation of the nerve roots (21). During the surgery, extensive flushing with physiological saline around the affected vertebra and manipulation disrupt the local microenvironment, thereby alleviating pain.

Although the posterior approach with pedicle screw fixation and bone graft fusion, with or without lesion clearance, has shown satisfactory results in the treatment of lumbar brucellar spondylitis, strict adherence to surgical indications is crucial for its clinical application. Based on the clinical data and surgical experience from this study, we summarize the indications for pure posterior spinal fixation and fusion as follows: (1) involvement of no more than 2 vertebral bodies without pedicle destruction or destruction less than 1/4; (2) the presence of a small abscess in the paravertebral or psoas muscle region; (3) small cavities or limited sequestrum formation in the bone destruction; (4) no obvious symptoms of spinal cord or nerve root compression. The indications for posterior spinal fixation plus lesion clearance through a posterior approach are: (1) severe destruction and collapse of the posterior column structure leading to spinal deformity and instability; (2) formation of abscesses within the spinal canal or intervertebral space; (3) significant destruction of the intervertebral space or intervertebral disc; (4) obvious compression of the spinal cord, nerve roots, or symptoms of cauda equina syndrome. The indications for posterior spinal fixation plus lesion clearance through an anterior approach are: (1) severe destruction of the anterior and middle column structures of the vertebral body and pedicle; (2) the presence of a large, difficult-to-absorb paravertebral or psoas muscle abscess; (3) significant destruction of the intervertebral space or intervertebral disc, without compression of the dural sac or nerve roots or protrusion into the spinal canal; (4) involvement of multiple segments of the vertebral body (≥3). Compared to conservative treatment, we observed that lesion clearance and fixation of the affected vertebral segment yielded significant improvements in VAS scores, ODI, complications, adverse drug reactions, laboratory examinations, radiographic evidence of bone fusion, neurological function recovery, and cure rate. This indicates that surgical treatment can reduce medication-related complications, improve efficacy, shorten the overall treatment duration, rapidly alleviate patients' back pain symptoms, and facilitate early functional activity. However, `it is still important to emphasize that drug therapy forms the foundation and essence of treatment, while surgery can expedite the healing process of the lesions. Therefore, in the selection of treatment for lumbar brucellar spondylitis, patient-specific factors should be comprehensively evaluated, and individualized treatment should be implemented. It is crucial not to solely pursue the non-invasiveness of drug therapy at the cost of prolonging medication duration and causing unnecessary harm to the body. Similarly, relaxing the surgical indications should not overlook the necessity and importance of drug therapy. It is essential to choose an appropriate surgical approach and minimize the potential harm to patients during surgery.

In summary, posterior vertebral fusion with bone graft fixation and posterior lesion clearance with bone graft fixation demonstrate faster and more effective outcomes compared to single-drug therapy. However, conservative treatment remains the preferred option for patients who meet the criteria for non-surgical treatment. Surgical intervention should be considered as a last resort for brucellar spondylitis. Nevertheless, when patients exhibit elevated levels of erythrocyte sedimentation rate (ESR), C-reactive protein (CRP), and localized infiltration depth observed through imaging, lesion clearance surgery should be performed.

The study has several limitations that should be acknowledged. It was conducted at a single center and had a small sample size, thus requiring a larger sample size and a multicenter study for validation. Secondly, the study design was retrospective, which may introduce confounding factors. Therefore, further exploration through prospective studies is necessary. Lastly, the study primarily focused on preoperative and intraoperative indicators related to bone tumor puncture and did not include long-term postoperative follow-up of tumor prognosis for patients in both groups.

Although the results of this study are satisfactory, there were some shortcomings. Firstly, this study was a retrospective single center case-controlled study with a low case study evidence level. Secondly, the study design was retrospective, which may introduce confounding factors. It only focused on comparing the efficacy of posterior spinal surgery with and without lesion clearance. The suitability of posterior surgery alone or combined anterior-posterior surgery for treating lumbar brucellar spondylitis remains uncertain. Therefore, it is necessary to conduct comparative analyses on larger sample sizes.

## Data Availability

The original contributions presented in the study are included in the article/Supplementary Material, further inquiries can be directed to the corresponding authors.
